# Neurogenic Bowel Dysfunction Changes after Osteopathic Care in Individuals with Spinal Cord Injuries: A Preliminary Randomized Controlled Trial

**DOI:** 10.3390/healthcare10020210

**Published:** 2022-01-21

**Authors:** Federica Tamburella, Alessandro Antonio Princi, Jacopo Piermaria, Matteo Lorusso, Giorgio Scivoletto, Marcella Masciullo, Giovanni Cardilli, Paola Argentieri, Marco Tramontano

**Affiliations:** 1 Santa Lucia Foundation IRCCS, Via Ardeatina 306, 00179 Rome, Italy; a.princi@hsantalucia.it (A.A.P.); jacopo.piermaria@gmail.com (J.P.); m.lorusso@hsantalucia.it (M.L.); g.scivoletto@hsantalucia.it (G.S.); giannicardilli75@gmail.com (G.C.); palestra.1@hsantalucia.it (P.A.); m.tramontano@hsantalucia.it (M.T.); 2 Centre pour l’Etude, la Recherche et la Diffusion Ostéopathiques “C.E.R.D.O.”, Via Magliano Sabina 23, 00199 Rome, Italy; 3 Neurology and Neurovascular Treatment Unit, Belcolle Hospital, Str. Sammartinese, 01100 Viterbo, Italy; m.masciullo@hsantalucia.it

**Keywords:** spinal cord injury, neurogenic bowel dysfunction, osteopathic manipulative treatment, constipation, swelling, enteric nervous system

## Abstract

Background: Neurogenic bowel dysfunction (NBD) indicates bowel dysfunction due to a lack of nervous control after a central nervous system lesion. Bowel symptoms, such as difficulties with evacuation, constipation, abdominal pain and swelling, are experienced commonly among individuals with spinal cord injury (SCI). Consequentially, individuals with SCI experience a general dissatisfaction with the lower perceived quality of life (QoL). Several studies have demonstrated the positive effects of manual therapies on NBD, including Osteopathic Manipulative Treatment (OMT). This study aimed to explore OMT effects on NBD in individuals with SCI compared with Manual Placebo Treatment (MPT). Methods: The study was a double-blind randomized controlled trial composed of three phases, each one lasting 30 days (i: NBD/drugs monitoring; ii: four OMT/MPT sessions; iii: NBD/drug monitoring and follow-up evaluation). Results: the NBD scale, the QoL on worries and concerns sub-questionnaire, and the perception of abdominal swelling and constipation significantly improved after treatments compared to baseline only for individuals who underwent OMT. Conclusion: These preliminary results showed positive effects of OMT on bowel function and QoL in individuals with SCI, but further studies are needed to confirm our results.

## 1. Introduction

The number of individuals suffering from central nervous system injury with bowel dysfunction is ever increasing. Spinal cord injury (SCI), both traumatic and non-traumatic, has an estimated prevalence of over 2.5 million worldwide [1] and among individuals with SCI, bowel symptoms are experienced commonly [2]: up to 95% report constipation and 75% have experienced episodes of faecal incontinence [3]. It is also recurrent for individuals with SCI to experience both constipation and faecal incontinence [4]. Moreover, difficulties with evacuation, constipation, abdominal pain and swelling are the most common symptoms [5].

The pathophysiology of neurogenic bowel dysfunction (NBD) is quite well-studied in individuals with SCI. There are two patterns of NBD after SCI: upper motor neuron bowel (UMN), which results from a spinal cord lesion above the sacral level, and the lower motor neuron bowel (LMN), which results from a lesion to the sacral spinal cord, roots or peripheral nerve innervation of the colon [6]. The LMN bowel represents a pattern of colonic dysfunction that results from a lesion of parasympathetic cell bodies at the conus, their axons in the cauda equina or the pelvic nerve. The LMN colon tends to be “relaxed”; no spinal cord-mediated reflex peristalsis occurs. The internal anal sphincter has an exaggerated smooth muscle response to rectal distention, which induces large rectal contractions. These contractions are associated with deep anal reflex and will most likely result in defecation without any increase in intra-abdominal pressure. On the other hand, the external anal sphincter is denervated, increasing the risk for incontinence [6]. The UMN bowel results from an SCI above the conus medullaris. UMN colon has been described as “spastic” because of the excessive colonic wall and anal tone observed. The striated external anal sphincter, normally under voluntary control, remains tight as a result of spasticity of the pelvic floor with consequent constipation. This condition necessitates a mechanical or chemical stimulus to trigger reflex defecation [6]. Furthermore, constipation is due also to the effects of medications (e.g., anticholinergics) and immobilization [4]. Other contributing factors are, e.g., loss of sensory function at the level of the rectum and perineum, incapability of active contraction of the pelvic floor muscles and variable loss of abdominal muscle contraction, hence the creation of intra-abdominal pressure. 

The experience of persons with SCI reveals that the risk and occurrence of both faecal incontinence and the difficulty with evacuation are particularly significant life-limiting problems [7]. Only 6% of individuals with SCI require no intervention to support their bowel function [8]. On the other hand, as many as 65% need to employ intrusive options [4], and one-third require assistance with bowel care. As a consequence of these conditions, in association with the clinical symptoms individuals with SCI experience loss of independence and dignity, embarrassment, anxiety, depression, social isolation, loss of sexual relationships and general dissatisfaction with the lower perceived quality of life (QoL) [9]. The burden of NBD is so great that individuals with SCI report bowel dysfunction among the most problematic conditions together with bladder dysfunction and sexual dysfunction, with a higher priority than pain, fatigue, perception of body image or walking [10].

For these reasons, rehabilitation goals in individuals with SCI should focus also on bowel care. The intervention consists of a comprehensive individualized person-centered bowel program, which may include diet, oral/rectal medications, equipment, physical intervention and scheduling of bowel care [6]. 

In the past years, several studies were carried out to evaluate the potential effects of manual therapies and gastrointestinal system dysfunctions [11]. One of these manual therapeutic approaches is the Osteopathic Manipulative Treatment (OMT), a whole-body intervention mainly focused on correcting the somatic dysfunctions (SDs) found in different regions of the body [12,13,14]. Osteopathic research has mostly been concerned with various clinical conditions such as musculoskeletal and neurological disorders [15,16,17,18,19]. The treatment of SDs using different osteopathic techniques can promote neurophysiological changes that presumably influence the neurovegetative system as reported in previous studies [14,20,21]. OMT effects on the gastrointestinal system were already investigated in different populations: women with constipation [22], people who suffer from Irritable Bowel Syndrome [23], children with anorectal malformation [24] and neurodevelopmental disorder [25]. These studies suggested OMT influence on visceral vascularization, physiological elasticity and visceral motility [23], and reported improvements in stool consistency, symptoms of constipation, the severity of constipation, and in reducing the use of laxative drugs. A recent study on the quality of life of women operated on for breast cancer and undergoing adjuvant chemotherapy showed that OMT, despite the fact that it does not reduce symptoms such as nausea and vomiting, improves symptoms related to constipation and reduces the use of anti-constipation drugs [26]. 

To date, there are no studies that have investigated the effects of OMT on bowel dysfunction in individuals with SCI. We hypothesize that OMT can facilitate visceral vascularization restoring the physiological elasticity/motility of the viscera and the peritoneal structures around the viscera [23] reducing the symptoms of NBD. For these reasons, this pilot study aims to explore the potential bottom-up effects of OMT on NBD in individuals with SCI, compared to a placebo group receiving standardized manual placebo treatment (MPT).

## 2. Materials and Methods

The study was conducted according to the guidelines of the Declaration of Helsinki and approved by the Independent Fondazione Santa Lucia Ethics Committee (protocol code CE/PROG. 800, date of approval 21 January 2020). Written informed consent was obtained from all participants according to the Fondazione Santa Lucia ethical procedures. The clinical trial was registered to ClinicalTrials.gov (NCT number: NCT04367571).

This study was a two-arm, double-blind randomized controlled trial with a 4-week follow-up. The study was composed of three phases, each one lasting 30 days: (i) observation and monitoring of NBD condition and drugs (Obs_pre); (ii) four OMT or MPT sessions with monitoring of NBD condition and drugs; (iii) observation and monitoring of NBD condition and drugs and follow-up evaluation (Obs_post). A researcher medical doctor who was not involved in the intervention sessions assessed the individuals’ eligibility to participate, based on the inclusion and exclusion criteria detailed below. Participants were randomly assigned to one of two groups: OMT group (OMTg) or MPT group (MPTg).

A consecutive sample of individuals with SCI admitted to the Neurorehabilitation 1 Department—Spinal Cord Unit of Fondazione Santa Lucia (a Research and Healthcare Institute) from April 2020 until August 2021 was recruited. 

The sample size was chosen in accordance with previous similar studies [27,28] —at least 13 patients should be included.

**Figure 1:**
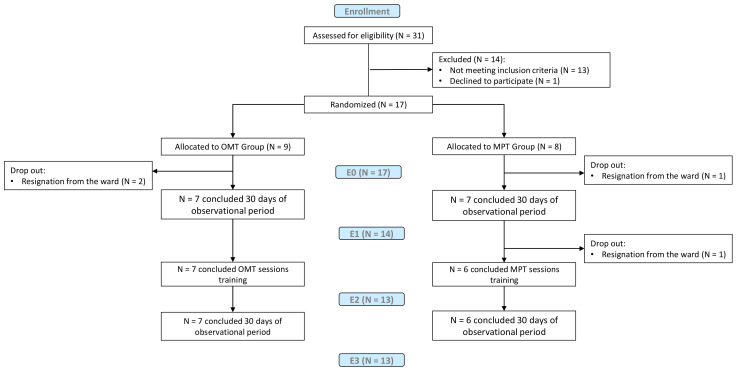
Flow chart of the study.

We excluded 13 individuals according to inclusion/exclusion criteria detailed below and 1 individual declined to participate (See Figure 1). The inclusion criteria were: (i) age between 18 and 70 years; (ii) SCI classified per the ASIA impairment scale (AIS) as AIS A, B, C or D; (iii) cervical or dorsal (up to D10) lesion;(iv) moderate or severe stable NBD condition per the Neurogenic Bowel Dysfunction Scale (NBDs); (v) no variations in the drug treatment plan during the Obs_pre period. Exclusion criteria were: (i) usage of bowel emptying techniques such as retrograde trans-anal irrigation; (ii) presence/previous inflammatory intestinal diseases; (iii) metabolic or endocrinological dysfunctions; (iv) pregnancy state; (v) cognitive disorders.

Individuals of both groups received 4 treatments, once a week for 4 weeks [22,23]. Each session lasted approximately 40 min. Individuals were not required to change their habits in terms of bowel management and were blinded to the received treatment. The OMT session was performed by healthcare professionals who completed osteopathy training program aligned with Italian Core Competencies in osteopathy [29] and with European Standard on Osteopathic Healthcare Provision. SDs were addressed according to tissue alteration, asymmetry, range of motion and tenderness parameters (TART), which guided the osteopathic evaluation and intervention (Educational Council on Osteopathic Principles) [30]. SDs were detected in the whole body, then balanced one by one to define a primary order of treatment according to TART parameters. For each participant, the osteopathic SOAP (subjective, objective, assessment, plan) note form was used, to record the body area evaluated and those treated. OMT techniques were focused on correcting the dysfunctions found during the initial physical examination and included myofascial techniques, balanced ligamentous tension, visceral manipulations and osteopathy in the cranial field [29,31,32]. The MPT was performed by the same osteopaths who carried out OMT and consisted of passive mobilizations of the pelvis, upper and lower limbs, cervical spine, and light manual touch on the abdomen and thoracic region. 

At enrollment (E0), after 30 observational days (E1), at the end of the 4 treatments (E2) and 30 days after last treatment (E3), individuals were evaluated according to the following primary and secondary outcome measures by a blinded researcher. 

Neurogenic Bowel Dysfunction Scale (NBDS) was selected as a primary outcome measure. The NBDS score is a 10 multiple-choice questionnaire developed for individuals with SCI and consists of symptom-based questions covering both constipation and faecal incontinence and their impact on QoL (subjective well-being, achievements) [4]. Higher total scores are representing more severe bowel dysfunction (0–6 very minor, 7–9 minor, 10–13 moderate, and 14 or more severe). After the original publication, a scale question was added on general satisfaction of the current bowel management ranging from 0 to 10 [33]. 

The Knowles Eccersley Scott Symptom Scale [34] (KESS) and the Patient Assessment of Constipation Quality Of Life (PAC–QOL) [35] questionnaire were selected as secondary outcome measures. The KESS is a validated questionnaire for the diagnosis of constipation and has the added advantage of differentiating between various subtypes of constipation. Items include the frequency of bowel movements using existing therapy, the difficulty of evacuation, laxative use, and time taken in the lavatory for bowel evacuation attempts. Total scores can range from 0 (no symptoms) to 39 (high symptom severity). A cut-off score greater than 11 indicates constipation. The PAC–QOL questionnaire is a brief but comprehensive tool, which evaluates constipation through a daily individual health assessment and functioning. This questionnaire consists of 28 self-reported items investigating the effects of constipation on the individuals’ QoL in the recent 2 weeks. The PAC–QOL questionnaire is subcategorized into 4 items on physical discomfort (PAC–QOL_physical), 8 items on psychosocial discomfort (PAC–QOL_ psychosocial), 5 items on treatment satisfaction (PAC–QOL_satisfaction), and finally 11 items on worries and concerns (PAC–QOL_worries). Response choice is a Likert scale from 0 to 4 (0 = none/not at all, 1 = a little bit/a little bit of the time, 2 = moderately/some of the time, 3 = quite a bit/most of the time, 4 = extremely/all the time). Higher scores mean higher negative effects on quality of life. An improvement (reduction) of ≥1 point in the PAC–QOL score was considered clinically significant based on previous validation studies.

Furthermore, before and after each single OMT or MPT session, individuals filled in three Visual Analogue Scales (VAS) [36] with a score ranging from 0 (minimum intensity) to 10 (maximum intensity) about abdominal pain (VAS_pain), perception of abdominal swelling (VAS_swelling) and intensity of perceived constipation (VAS_constipation) [34]. 

During the study, for each individual, the number of incontinence events and the number of daily bowel movements (spontaneous or after administering an enema), with the related stool consistency per the Bristol Stool Chart [37] (BSC), were recorded by the nurse staff on a daily bowel diary. BSC is a medical aid designed to classify stools into seven categories (type 1–2 indicate constipation, type 3–4 are ideal stools as they are easier to pass, and type 5–7 may indicate diarrhea). The number of bowel movements, both spontaneous or not, were recorded and reported as % of the total number of evacuations across the 3 months, as well as the % of incontinence episodes during the 30 days of treatment compared to the observational period, and the follow-up was registered. In the daily bowel diary, the type and dosages of each drug were reported by nurse staff for the 3-month periods (Obs_pre, treatment and Obs_post) according to different categories: oral laxative (powder, compress or syrup), rectal laxative, enema.

An independent person who was not responsible for determining the eligibility of individuals carried out the randomization. Block randomization was performed with a computer-generated randomization list using a block size. The researcher responsible for the randomization process deposited the list in secure web-based storage. Following the initial assessment, the participant was given a sealed envelope, prepared by a research assistant, containing their allocated intervention group. 

As concern statistical analysis, descriptive statistics were generated for all variables. Primary and secondary outcome measures baseline data (E0) of OMTg and MPTg were compared per the Mann–Whitney U test. For non-parametric data (NBDS, KESS, PAC–QOL, PAC–QOL_physical, PAC–QOL_ psychosocial, PAC–QOL_satisfaction and PAC–QOL_worries) the Kruskal–Wallis Test was used to compare data collected at different assessment time points (E0, E1, E2, E3) for both OMTg and MPTg. For these data the effect size (ES) was calculated per the eta squared (small ES: for η^2^ between 0.01 and 0.06, moderate ES: for η^2^ between 0.06 and 0.14, large ES ze: η^2^ ≥ 0.14). Paired *t*-test was selected to compare VAS_pain, VAS_swelling and VAS_constipation data collected before the first treatment (T1) vs. data collected after the last treatment (T4) for each group, OMT or MPT one. For these data the ES was calculated per the Cohen’s *d* (*d* = 0.2 is considered a small ES, *d* = 0.5 represents a medium ES, *d* = 0.8 a large ES, *d* = 1.3 a very large ES).

Statistical significance was set at *p* < 0.05. Statistical tests were performed using the Statistical Package for the Social Sciences Software (SPSS), version12.0 (Chicago, IL, USA).

## 3. Results

A total of 31 individuals with SCI were initially recruited. Thirteen individuals were excluded according to the inclusion/exclusion criteria detailed above, while one individual declined to participate. The 17 individuals included were randomized into OMTg (N = 9) or MPTg (N = 8) (See Figure 1). 

The full cohort of 17 individuals completed E0 assessments. During the Obs_pre period, two individuals from OMTg and one from MPTg dropped out because of discharge from the hospital. Consequently, 14 individuals performed E1 assessments and started the treatments. One participant was discharged after two MPT sessions. Therefore, 13 individuals underwent E2 and E3 assessments (see Figure 1). No adverse effects due to OMT or MPT were reported for any individual.

At E0, no clinical-based assessments differed significantly between OMT and MPT groups (*p* > 0.05). Epidemiological and neurological data of enrolled individuals are described in Table 1.

No significant differences between E0 and E1 data were pointed out for any measure, suggesting a stable NBD condition for both OMTg and MPTg before starting training sessions. By statistical comparison, it showed that significant differences across evaluation time points (E0, E1, E2, E3) were obtained only for OMTg as detailed below.

### 3.1. Primary Outcome Measure

**Figure 2:**
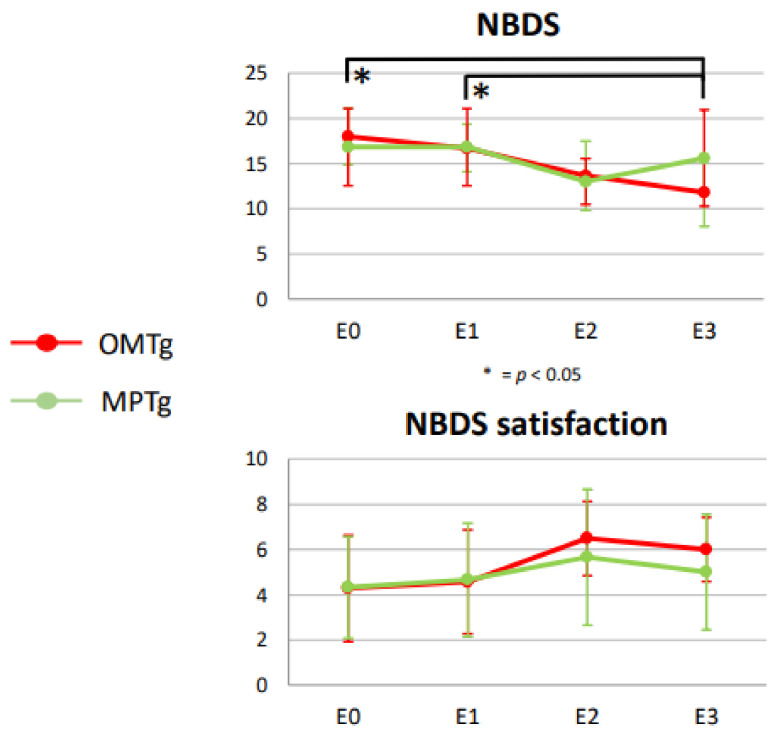
Neurogenic Bowel Dysfunction (NBD) scale results collected at E0, E1, E2, E3 were reported for both Osteopathic Manipulative Treatment (OMT) (red lines) or Manual Placebo Treatment (MPT) (green lines) groups; * = *p* < 0.05.

The NBDS score was significantly improved only in the OMTg in the comparison E0 versus E3 (*p* = 0.011) and E1 versus E3 (*p* = 0.040) with respectively a small (η^2^ = 0.0036) and a moderate ES (η^2^ = 0.013). The NBDS question about the general satisfaction of the bowel management showed a positive trend more evident for the OMTg (see Figure 2). 

### 3.2. Secondary Outcome Measures

**Figure 3:**
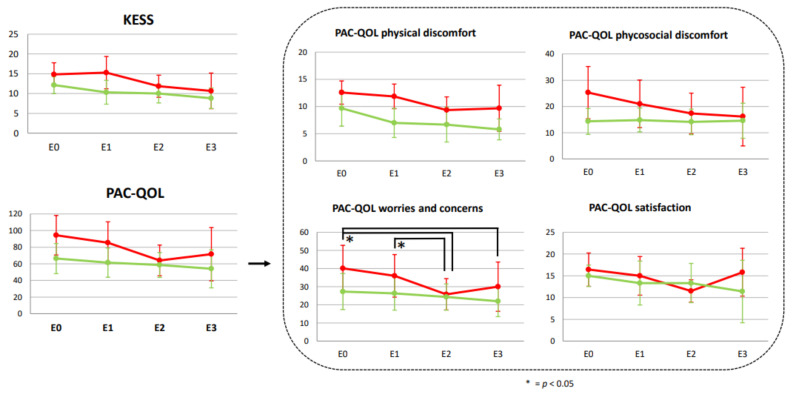
Knowles Eccersley Scott Symptom (KESS) scale and the Individual Assessment of Constipation–Quality of Life (PAC–QOL) questionnaire results collected at E0, E1, E2, E3 were reported for both OMT (red lines) or MPT (green lines) groups; * = *p* < 0.05.

KESS score improvement was better in the OMTg in the comparison E1 versus E2 with no further improvements at follow-up examination (*p* > 0.05). MPTg did not allow any variations in the 3 months (*p* > 0.05) (see Figure 3).

PAC–QOL_worries score in the comparison E1 versus E2 (*p* = 0.029) as well as in the comparison E0 versus E2 (*p* = 0.031) or E3 (*p* = 0.041) (see Figure 3) was significantly improved only in the OMTg, with moderate ES (η^2^ respectively equal to 0.010 and 0.0136). QoL related to “worries and concerns” and “psychosocial discomfort” showed an improvement in OMTg (see Figure 3).

**Figure 4:**
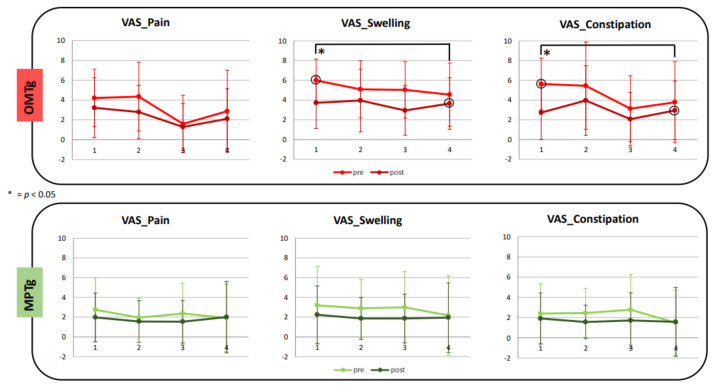
Visual Analogue Scales (VAS) results about individuals’ perception of pain, swelling and constipation before (pre) and after (post) each OMT or MPT session were reported for both OMT (red lines) or MPT (green lines) groups; * = *p* < 0.05.

VAS daily score showed significant improvements in terms of reduction of the sense of constipation (*p* = 0.031) and mainly swelling (*p* = 0.006) (see Figure 4), in comparison to the evaluation performed before the first OMT session, with a large ES respectively of *d* = 0.99 and *d* = 0.83. In addition, VAS_pain was reduced after OMT, even if it was not significantly. No statistical changes were observed in the VAS score in the MPTg.

**Figure 5:**
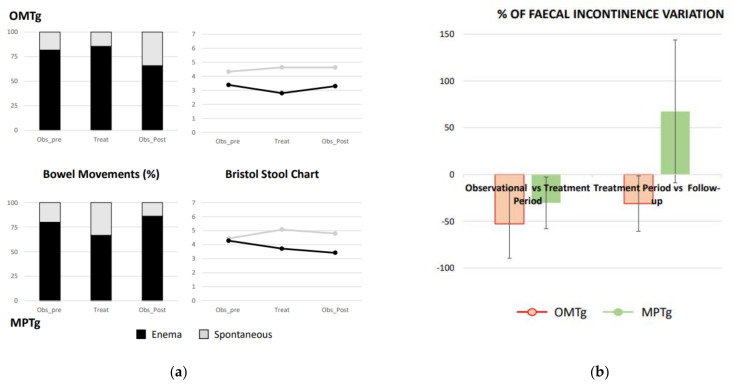
Daily bowel diary data. (**a**) Spontaneous bowel movements (%—grey columns) or bowel movements after administering enema (%—black columns) are reported with the related Bristol Stool Chart (BSC) score; (**b**) % of incontinence episodes variation during the treatment period compared to the observational period before OMT or MPT sessions and to the follow-up one for the OMT group (OMTg) (red columns) and MPT group (MPTg) (green columns). “-“ refers to a reduction into faecal incontinence, while “+”refers to an increment into faecal incontinence.

Bowel movements, spontaneous or after administering an enema, are similar between OMTg and MPTg at E0. There was a prevalence of bowel movements resulting from administration of enemas, compared to spontaneous evacuations. For people who underwent OMT during the treatment period, the number of bowel movements after enemas slightly increased, while in the follow-up the situation appears to be reversed compared to the baseline. For the MPTg the pattern was reversed with an increase in evacuations resulting from enema treatment in the follow-up (see Figure 5a). Data relating to the BSC do not show changes during the study for either group (see Figure 5a).

Faecal incontinence episodes during the treatment period were reduced compared to baseline for either OMTg or MPTg. An opposite trend between groups was pointed out in the comparison between faecal incontinence episodes during the treatment period versus the follow-up. In the OMTg, the benefits observed during the treatment period continued after the treatment. Conversely, for the MPTg there was a tendency to worsen after training interruption (see Figure 5b).

During the study period, no variations in the drug assumption were reported in both groups. Only exceptions were observed for two individuals who underwent OMT and for 1 individual of the MPTg (Table 2). 

### 3.3. OMT Treatment Data

**Figure 6:**
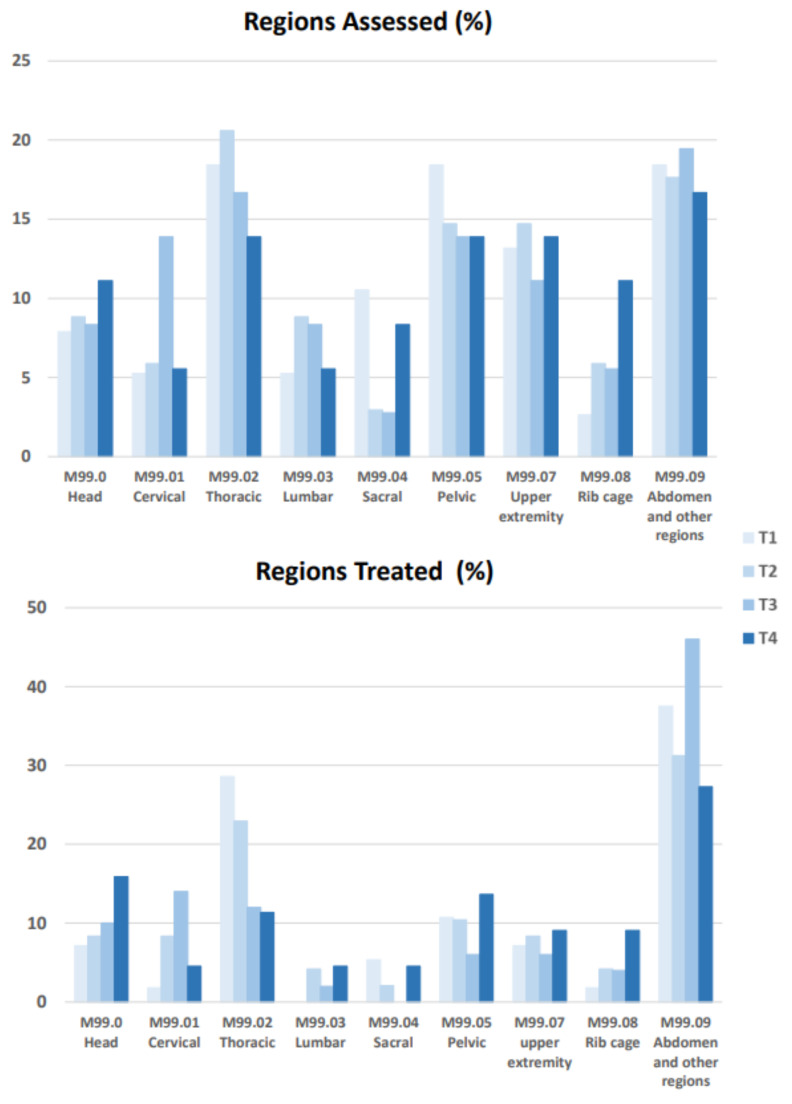
Localization of treated and assessed somatic dysfunctions reported for the different body regions according to somatic dysfunction classifications in OMTg.

Only OMTg, details about body area evaluated and treated were collected and reported (%), for each treatment (T1, T2, T3 and T4). The regions with the high percentage of SD were the abdomen, pelvis and thorax (see Figure 6). According to the osteopathic assessment, the treatment was mainly focused on the abdomen and thoracic regions, followed by the pelvic one. Across time, the number of dysfunctions treated in the thoracic area gradually decreased, while the abdomen remained the most treated area. Nevertheless, the comparison between T1 and T4 showed a reduced number of treated abdominal SD. The treatment of the head and the rib cage were similar, with a progressive increase of the treated dysfunctions across time. The trend of the cervical region was similar, with the exception that at T4 the treated SD were drastically reduced in comparison to T3. The less-treated areas were the lumbar and sacral ones.

**Figure 7:**
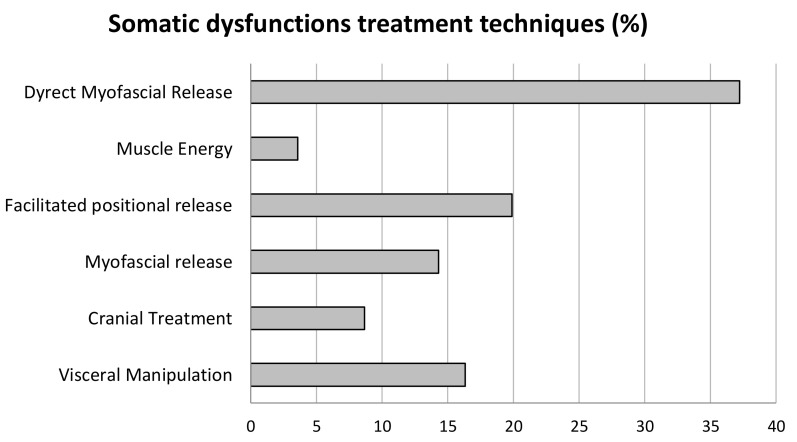
Techniques selected for the treatment of the different somatic dysfunctions in OMTg (%).

The most-used technique was the direct myofascial release, followed by the facilitated positional release and visceral manipulation. The less-used techniques were those based on muscle energy (see Figure 7). 

## 4. Discussion

This study aimed to explore the effects of OMT on NBD symptoms in individuals with SCI, compared with MPT. Disorders of bowel function in individuals with SCI depend on both neurological factors related to the injury and factors related to immobilization and lifestyle. These elements favor the onset of extremely prolonged intestinal transit and, decreased colon motility that result in frequent manifestations of abdominal distension, constipation accompanied by symptoms such as bloating, pain and abdominal constipation [38]. The starting hypothesis of the study was based on the assumption that OMT may influence the central nervous system [20,21], autonomic nervous system [14], haemodynamic system [14] and visceral motility [23] as reported in previous clinical studies [22,23,24,25]. 

Our results showed an improvement in the NBDS scores only in the OMTg after the treatment. The NBDS is considered the most comprehensive tool for NBD assessment, and it has strong psychometric properties and responsiveness to change in QoL due to modifications in bowel function [4]. Interestingly, this improvement was significant and with a small or moderate effect size, respectively, in the comparison E0 versus E3 and E1 versus E3, suggesting the long-lasting effects of OMT in improving bowel function (see Figure 2). Starting from a stable NBD condition associated with no changes in the drug treatment plan, it can be assumed that this modification could be related to a bottom-up effect of OMT. In support of this hypothesis, no significant changes were found in the MPT group for the primary outcome measure. Accordingly, the results about QoL showed no significant PAC–QOL, questionnaire variations for MPTg. Instead, OMT allowed a general trend of benefit in QoL after treatments. It is intriguing that at the end of the 4 sessions all the items investigated by the PAC–QOL improved, mainly those exploring the worries and concerns (see Figure 3). In the following month (Obs_post), a reversal of the trend was observed for worries/concerns and satisfaction subcategories without ever reaching the initial levels of apprehensions or dissatisfaction. This trend could be explained by the fact that individuals with SCI are aware of being at the end of the training, thus increasing their concerns for the future and the management of bowel conditions. Consequently, this could affect the level of satisfaction. Interestingly, this happens only for the OMTg, demonstrating that the benefits derived from OMT were directly perceived by individuals with SCI. Indeed, the MPTg did not show changes between the baseline and the end of the treatment. These results could demonstrate that NBD directly affects the perceived QoL due to SCI [9]. This result is crucial because the impact of poor bowel management extends far beyond impaired intestinal motility: fear of bowel accidents is a commonly stated reason why persons with SCI do not engage in activities outside of the home or travel away from home [6]. Furthermore, comparing VAS data between T1 and T4, results showed a significant reduction and a large effect of individuals’ perception of swelling and constipation only for the OMTg (see Figure 4). These results also indicate that people with SCI perceive the effect of the OMT in these terms. 

For two individuals who underwent OMT, an interruption of the drug (oral laxatives or enemas) during the month of treatment or the next one (Obs_post) was recorded. These improvements, expressed as drug withdrawal, were maintained even in the absence of the treatment itself. On the contrary for the remaining OMTg individuals, no changes were reported. Moreover, for the MPTg no notable changes were observed for the entire treatment period compared to the baseline (see Table 2). 

Speculating on these results, we can hypothesize a possible relationship between OMT and the effects evidenced. “Neurogenic bowel” is a term that relates bowel dysfunction to a lack of nervous control [6]. Patterns of dysfunction are described in relation to neural lesions located within the central nervous system, the peripheral nerves, and the enteric nervous system (ENS). Interestingly, after SCI ENS remains functionally intact local synaptic remodeling would be expected to occur in response to spinal lesions, but the effects on enteric gut nervous control, if any, are unknown [6]. On the contrary, it is well known that ENS can work completely independently of any neural input from the central nervous system [39]. The most local neurogenic mechanism of colonic control comes through the ENS, which coordinates all segmental motility and some propagated movement operating independently within the colonic wall. This colo-colonic intramural reflex has become known as “the law of the intestine” [6]. Whenever the intestinal wall is stretched or dilated, the nerves in the myenteric plexus cause the muscles above the dilation to constrict and those below the dilation to relax, propelling the contents caudally. This intramural wiring facilitates bolus transfer. This ENS control of the colonic wall is modulated through central connections from parasympathetic and sympathetic systems (i.e., neurogenic control of colonic mobility [40]). OMT has a role in modulating the autonomic nervous system [41]; indeed, we can hypothesize that it can influence the neurogenic mechanism of colonic control through the ENS function modulation allowing an effect in patients who suffer from NBD. Furthermore, taking into account the partial or complete absence of control/action of the higher centers, the action of the OMT could be concentrated also at the level of the effector organs [42,43]. 

According to a recent review [44], the OMT techniques selected by practitioners [14,21] highlighted a preponderance of soft techniques, such as direct myofascial release, facilitated positional release or visceral manipulation (see Figure 7). It is interesting to note, besides the selected techniques, that the most treated regions were the abdomen and thoracic ones. Even if across time the number of SDs treated in these regions gradually decreased, this one remained the most treated region. Nevertheless, pelvis and head also were treated for a long time. In light of the primary outcome measure results and the OMT beneficial effects on QoL, the combination of these data suggests that myofascial and visceral manipulation techniques, applied to the above regions, may allow visceral vascularization and can restore the physiological elasticity and mobility of the viscera [25] with a consequent reduction in the bowel-related symptoms. Future studies could be aimed at confirming these data and also could compare the effects of different techniques on OMT effectiveness in patients with NBD. Besides the possible OMT on ENS function modulation in patients suffering from NBD, OMT treatment was focused also on regions influenced by prolonged sitting. In fact, the pelvis and upper limbs regions also were treated (see Figure 6). This could be linked to the forced and prolonged position that individuals maintain every day in the wheelchair and to the overuse of the upper limbs for the management of postural transfers and the activities of daily life. 

This study has some limitations that should be considered when interpreting the results, such as the sample size and the heterogeneity of the sample enrolled, but this is to be considered as a pilot study. Nevertheless, the significance of the NBDS, PAC–QOL_worries, VAS_swelling and VAS_constipation scores was associated with an effects size ranging from low to large, suggesting the potential practical significance of these results. Furthermore, as suggested by Friston [45], significant results that are based on a small cohort might indicate a larger treatment effect than equivalent results in a larger sample. Results of this study allow a better design for further study on this topic, including individuals with SCI with more homogeneous features and instrumental evaluations of bowel function. For instance, the introduction of the colonic transit time per the RX techniques could help improve understanding of the OMT effects on individuals with SCI. Lastly, the recruitment of individuals with SCI was affected by the COVID-19 pandemic.

## 5. Conclusions

This was the first study aimed at investigating the effects of OMT on NBD in individuals with SCI.

Our preliminary results showed positive effects due to OMT on bowel function, and worries and concerns related to NBD, as well as on the perceived swelling and constipation. No significant improvements were reported for KESS score, PAC items related to physical and psychosocial discomfort, or satisfaction and perceived abdominal pain.

## Figures and Tables

**Table 1:** Epidemiological and neurological data of enrolled individuals.

	Individuals	Age (sd)	Sex	Lesion Level	Time Since Injury (Months)	AIS	Aetiology
OMTg	*1*	*25*	*M*	*C4*	*7.5*	A	Traumatic
	*2*	*37*	*M*	*C6*	*19.5*	D	Traumatic
	*3*	*51*	*M*	*D10*	*19.5*	A	Traumatic
	*4*	*31*	*M*	*T6*	*6*	A	Traumatic
	*5*	*28*	*M*	*C5*	*2*	C	Non Traumatic
	*6*	*54*	*M*	*C3*	*1*	C	Traumatic
	*7*	*37*	*F*	*D10*	*1*	D	Non Traumatic
	*Mean*	*37.5 (11,1)*	*6M-1F*		*8.16*		
MPTg	1	*66*	*M*	*C4*	*6*	B	Traumatic
	*2*	*51*	*M*	*D9*	*5*	C	Non Traumatic
	*3*	*69*	*M*	*D10*	*9*	D	Non Traumatic
	*4*	*18*	*M*	*D10*	*19*	A	Traumatic
	*5*	*77*	*F*	*D7*	*3.5*	D	Non Traumatic
	*6*	*35*	*M*	*D5*	*3.5*	A	Non Traumatic
	*Mean*	*52.6 (22,5)*	*5M-1 F*		*7.66*		

**Table 2:** Drug treatment plan during the Obs_pre, treatment and Obs_post periods for OMT e MPT groups.

		Obs_Pre					Treatment					Obs_Post				
		Oral Laxative			Rectal Laxative	Enema	Oral Laxative		Rectal Laxative		Enema	Oral Laxative			Rectal Laxative	Enema
	Ind.	Powder	Compr.	Syrup			Powder	Compr.	Syrup			Powder	Compr.	Syrup		
OMTg	***1***	2				2	2				2	2				2
	***2***		2	7		2		2	7		2		2	7		2
	***3***	2		7		2	2		7		2	2		7		2
	***4***					3					3					3
	***5***	2				2	2				2	2				2
	***6***	3				3	3				3	3				3 #
	***7***		2			3		2 #			2 #					
MPTg	***1***	2		7	3	2	2		7	3	2	2		7	3	2
	***2***	2				2	2				2	2				2
	***3***	2		7		3	3 *		7		3			7		3
	***4***					3					3					3
	***5***	2				2	2				2	2				2
	***6***	7		7		2	7		7		2	7		7		2

Frequency of administration is reported as times per week (* refers to a change in the drug treatment plan with respect to the period before; # indicates that the drug was suspended after the first half of the reporting period; Compr: compress; Ind.: individuals with SCI).

## Data Availability

The data presented in this study are available on request from the corresponding author.
